# Evaluation of agreement between a noninvasive method for real-time measurement of critical blood values with a standard point-of-care device

**DOI:** 10.1371/journal.pone.0304706

**Published:** 2024-06-18

**Authors:** Rudi H. Ettrich, Joshua Caballero, Prashant Sakharkar, Sultan Ahmed, Traci Hurlston, Jayesh Parmar, Subrata Deb

**Affiliations:** 1 College of Biomedical Sciences, Larkin University, Miami, FL, United States of America; 2 Wertheim College of Medicine, Florida International University, Miami, FL, United States of America; 3 College of Pharmacy, University of Georgia, Athens, GA, United States of America; 4 College of Pharmacy, Larkin University, Miami, FL, United States of America; Chunghwa Telecom Co. Ltd., TAIWAN

## Abstract

The purpose of this work was to investigate the degree of agreement between two distinct approaches for measuring a set of blood values and to compare comfort levels reported by participants when utilizing these two disparate measurement methods. Radial arterial blood was collected for the comparator analysis using the Abbott i-STAT^®^ POCT device. In contrast, the non-invasive proprietary DBC methodology is used to calculate sodium, potassium, chloride, ionized calcium, total carbon dioxide, pH, bicarbonate, and oxygen saturation using four input parameters (temperature, hemoglobin, pO2, and pCO2). Agreement between the measurement for a set of blood values obtained using i-STAT and DBC methodology was compared using intraclass correlation coefficients, Passing and Bablok regression analyses, and Bland Altman plots. A p-value of <0.05 was considered statistically significant. A total of 37 participants were included in this study. The mean age of the participants was 42.4 ± 13 years, most were male (65%), predominantly Caucasian/White (75%), and of Hispanic ethnicity (40%). The Intraclass Correlation Coefficients (ICC) analyses indicated agreement levels ranging from poor to moderate between i-STAT and the DBC’s algorithm for Hb, pCO2, HCO3, TCO2, and Na, and weak agreement for pO2, HSO2, pH, K, Ca, and Cl. The Passing and Bablok regression analyses demonstrated that values for Hb, pO2, pCO2, TCO2, Cl, and Na obtained from the i-STAT did not differ significantly from that of the DBC’s algorithm suggesting good agreement. The values for Hb, K, and Na measured by the DBC algorithm were slightly higher than those obtained by the i-STAT, indicating some systematic differences between these two methods on Bland Altman Plots. The non-invasive DBC methodology was found to be reliable and robust for most of the measured blood values compared to invasive POCT i-STAT device in healthy participants. These findings need further validation in larger samples and among individuals afflicted with various medical conditions.

## Introduction

Blood gas measurement is a monitoring or diagnostic test that is used to determine the levels of partial pressures of oxygen (pO2) and carbon dioxide (pCO2) circulating in the blood [1]. These values can be used to calculate several biomedical parameters including bicarbonate levels and electrolytes [2, 3]. In humans, pO2 and pCO2 can be measured either by invasive or non-invasive techniques. Invasive methods involve obtaining a sample of blood directly from an artery, such as the radial artery in the wrist, while non-invasive methods, primarily, use transcutaneous devices to estimate the oxygen and carbon dioxide levels via skin [4]. The arterial blood gas (ABG) requires specialized devices and expertise to analyze the results. In contrast, transcutaneous non-invasive methods do not involve the withdrawal of blood samples and use sensors that are placed on the skin to measure the amount of oxygen and carbon dioxide in the blood. Blood gas measurements can be used to assess the acid-base balance and to detect respiratory or metabolic disorders of the patient, especially, in emergency situations [5]. The results of the blood gas measurements are used to determine the oxygenation status and to guide treatment decisions, such as the use of oxygen therapy, mechanical ventilation, or pharmacological interventions.

Transcutaneous devices employ sensors that are placed on the skin (e.g., arms, forehead, earlobe) to measure pO2 and pCO2 levels. Interestingly, the technology in the sensors for transcutaneous devices is fast evolving. For example, oxygen diffusion and pH-sensitive changes in voltage are used in transcutaneous devices [6]. As oxygen diffuses through the membrane, it causes a change in the LED’s light intensity which is then detected by the photodiode. The transcutaneous pO2 levels are measured using the principle of oxygen diffusion where the sensor is made up of a membrane that is permeable to oxygen and has a light-emitting diode (LED) on one side and a photodiode on the other side [4, 7]. Similarly, transcutaneous pCO2 measurement uses the principle of pH changes caused by the presence of CO2. The sensor is composed of a pH-sensitive electrode which changes its voltage as per the skin pH and this voltage is then converted to pCO2 [4]. These sensors subsequently utilize this information to calculate the amount of O2 or CO2 in the blood.

The use of algorithms to calculate different blood values from pO2 and pCO2 is a common approach in blood gas measurements. Mathematical equations are used to calculate and convert arterial or transcutaneous oxygen and carbon dioxide measurements into electrolyte and mineral levels. One example of an algorithm used for this purpose is the Stewart approach which includes pCO2, bicarbonate level and pH in the equation [8]. Similarly, base excess (BE) algorithm derived from Siggaard-Andersen equation involves pCO2 and pH values to calculate the metabolic component of the acid-base balance in the blood and detect metabolic acidosis or alkalosis [9, 10]. The Fick’s principle is employed to derive oxygen content in the blood using pO2 and hemoglobin values [11]. The accuracy of these algorithms may differ based on the physiology of the patient, the device used, and the environment [12].

In emergency settings, blood draws are performed to assess critical values and optimize care. There are several point-of-care testing (POCT) devices for measuring critical laboratory values, but their real-time utility can be cumbersome considering need for blood draws, requiring using multiple cartridges, and thus potentially delaying getting results on time. Digital Blood Corporation (DBC; Fort Lauderdale, FL) developed and successfully patented [13] a non-invasive POCT system that can measure patients’ set of blood values in real-time ‘using four user-input sensors. Furthermore, it can be used for continuous monitoring of a patient’s blood environment parameters. Abbot i-Stat is a portable blood analyzer that delivers lab-quality, diagnostic results quickly. In the United States this invasive POCT device is most widely used in clinical set up and is considered the “gold standard” among the available POCT devices. Thus, i-STAT was chosen for direct comparison with DBC’s non-invasive approach. The primary objective of our study was to (a) to investigate the degree of agreement between non-invasive proprietary DBC algorithm and the well-established invasive i-STAT POCT device, as a reference standard and (b) to conduct a comparative analysis of the comfort levels reported by participants when utilizing these two distinct measurement approaches.

## Materials and methods

### Study design and methods

Our study was carried out in two sequential phases. The initial phase of the study was designed as a pilot investigation. During this phase, a relatively small sample of only healthy subjects was selected as participants to perform refinement and validation of the DBC algorithm and software. In line with best practices for a study of this type, we deliberately did not include ill subjects to protect them from any potential harm of using DBC and a comparator i-STAT device. The primary focus was to ascertain the level of agreement between the i-STAT device and the DBC algorithm and software, employing the four non-invasive measurements as input parameters. This preliminary phase served as a testing ground for methodological refinement and validation.

The subsequent phase of the study was designed to include a significantly larger and more diverse sample, comprising both healthy individuals and those afflicted with various medical conditions. This phase aimed to evaluate the accuracy and precision of measurements provided by both the i-STAT device and the DBC algorithm and software. Furthermore, it sought to validate and extend the assessment of agreement between these two methods across a broader spectrum of participants using individuals with diverse health profiles.

### Inclusion and exclusion criteria for the subjects

A cohort of healthy adult individuals ranging from 18 to 70 years of age, either spoke English or Spanish language, and seeking ambulatory healthcare services at one medical clinic in Broward County, Florida during June to November 2021 were included as subjects.

Individuals diagnosed with hematological disorders such as hemophilia and uncontrolled coagulation disorders were excluded. Additionally, individuals on anticoagulant therapy, including warfarin, low molecular weight heparins (such as enoxaparin), direct thrombin inhibitors (dabigatran), or factor Xa inhibitors (e.g., apixaban), were also excluded from participation, with the only exception being on a daily dose of Aspirin not exceeding 325 mg. The recruitment period for this study encompassed from June 1, 2021, to November 30, 2021.

The study was approved by the Institutional Review Board of Larkin University, and all adult participants provided written informed consent for their participation. No minor participants were included in this study.

### Institutional review board statement

The study was conducted according to the guidelines of the Declaration of Helsinki and approved by the Institutional Review Board (or Ethics Committee) of Larkin University (protocol code COP052621-01F and date of approval May 26, 2021).

### Analyzers employed

#### Abbot i-STAT POCT device (invasive)

Abbott i-STAT POCT device was used in this study as a reference standard. This portable device is engineered to conduct assessments of blood gases, electrolytes, metabolites, and coagulation parameters. It achieves this through the utilization of single-use i-STAT test cartridges, which encompass an expansive menu of diagnostic assays, all integrated into a singular, portable platform. Each individual test cartridge is distinguished by its unique combination of biosensors, tailored to cater to an array of clinical needs. This unified testing system removes the necessity for multiple distinct analyzers. The analytical performance of the i-STAT POCT device has been validated in several human studies attesting to its accuracy and reliability in the measurement of blood gases and electrolytes [14–16].

#### Proprietary DBC methodology (non-invasive)

The DBC (Digital Blood Corporation) algorithm and accompanying software constitute a computational system engineered for the analysis of acid-base and ionic equilibrium parameters within blood gases, employing a non-invasive transcutaneous methodology. These algorithmic components are integrated within a computing unit block and interface with analog and/or digital input sensors. These sensors are responsible for measuring critical parameters, including pO2, pCO2, hemoglobin levels in the patient’s blood, and temperature. The values obtained from these individual sensors are subsequently transmitted to complex mathematical algorithms. Here, any alteration in a measured input value from each sensor independently influences the values pertaining to blood gases and measured ions, providing real-time changes with the highest degree of precision. The algorithm calculates values for eleven essential parameters, including sodium, potassium, chloride, ionized calcium (Ca), total carbon dioxide (TCO2), pH, bicarbonate, and oxygen saturation (SO2) by utilizing these four input values (DBC US patent).

### Blood collection and transcutaneous measurements

For the assessment of blood gases and electrolytes, the Abbott i-STAT® POCT device employs EG7+ and CHEM8+ cartridges. Whereas the Masimo Rad-57 device is utilized for the measurement of temperature and hemoglobin levels. Simultaneously, the TINA TCM4 radiometer is employed to measure partial pressures of oxygen (pO2) and carbon dioxide (pCO2), generating the requisite input values for the DBC’s algorithm. The Masimo RAD 57 device employs multiple light wavelengths to measure parameters such as total hemoglobin accurately and noninvasively, oxygen content, carboxyhemoglobin, and methemoglobin continuously. In parallel, the TINA TCM4 radiometer serves as a non-invasive transcutaneous monitoring device, catering to the needs of patients requiring continuous monitoring of oxygen and carbon dioxide levels with minimal blood draw. The DBC algorithm, therefore, leveraged these instruments to obtain measurements for the parameters of interest.

### Assessment of comfort level

The assessment of participants’ comfort levels was measured through the self-administered questionnaire consisting of six items. These items targeted the emotional state, uneasiness with attachment, perception of harm, perceived changes, movement constraints, and anxiety level. Participants were asked to rate their comfort levels on a scale ranging from "0" (indicating low comfort) to "10" (indicating high comfort) independently after the completion of measurements using both the i-STAT device and the DBC algorithm (S1 Fig).

### Data analysis

We subjected data to the Shapiro-Wilks test to assess normality. In instances where the normality assumption was not met, a logarithmic transformation was applied to the data. The data were analyzed for descriptive statistics. Continuous variables were presented as mean with standard deviations (SD), while categorical variables were expressed as frequencies and percentages. To assess the variation within subjects for measurements obtained using the DBC algorithm, the coefficient of variation (CVw) was calculated. Comparisons between the i-STAT device and the DBC algorithm were carried out using paired t-test or Wilcoxon sign rank test, to assess the agreement between the two methodologies [17]. To quantitatively assess the degree of agreement between the i-STAT device and the DBC algorithm, the Intraclass Correlation Coefficients (ICC) were calculated. The ICC serves as a vital indicator of concordance between the two measurement techniques, yielding scores that range from 0 (indicating no agreement) to 1 (indicating perfect agreement). For this study, we used a single-measure two-way mixed absolute model, where the method effect was treated as fixed and the participant effect as random. The ICC values below 0.5 signifies poor agreement, values between 0.5 and 0.75 indicate moderate agreement, values ranging from 0.75 to 0.9 denotes good agreement, and any ICC value of 0.9 and above indicates excellent agreement. Like the Pearson’s correlation coefficient, the ICC is also influenced by the degree of heterogeneity present in the sample population [18].

Additionally, we used Passing and Bablok regression analysis to assess the agreement between these two analytical methods and identify any potential systematic bias [19]. This method is particularly robust and non-parametric, thus making it insensitive to the distribution of errors and the presence of data outliers. Moreover, it operates under the assumption that measurement errors in both methods exhibit similar distributions, which need not necessarily conform to normality. It accommodates variances that maintain a constant ratio, acknowledges arbitrary sampling distributions, and considers imprecision.

The intercept A and the slope B in Passing and Bablok regression analysis quantifies and measures the systematic and proportional differences between the two methods, respectively. To determine whether there exists a statistically significant difference, the hypotheses of no difference are accepted if the confidence interval for "A" contains the value 0 and the confidence interval for "B" encompasses the value 1. This analysis provides valuable insights into the presence of any systematic deviations or proportional biases between the analytical methods to evaluate agreement [20].

To assess the congruence between the DBC algorithm and the reference method (i-STAT), we used the Bland and Altman (B&A) plot, a well-established tool for examining agreement between two measurement techniques [21]. This analytical approach allows to calculate both the absolute and relative differences, as well as determine the limit of agreement between the two methods [21–23]. The Bland and Altman method suggests that approximately 95% of the data points should fall within the range of ±2 standard deviations (2s) from the mean difference. In the realm of clinical chemistry, the B&A plot serves as a widely accepted means to quantitatively evaluate the concordance between two quantitative measurements, particularly in instances where traditional correlational analysis methods proven less appropriate.

While the B&A plot method effectively describes the intervals of agreement, it does not inherently establish whether these limits are deemed acceptable or not. The determination of acceptable limits should be based on a priori definition, rooted in clinical necessity, biological considerations, or other pertinent objectives. In essence, the appropriateness of the observed limits of agreement should be predicated on the specific context and goals of the investigation [22]. In this study, the B&A limit of agreement were compared with the acceptable clinical limits for parameters suggested by Ricos et al. and Westgard QC [24]. The sample size of 25 participants for ICC analyses based on degrees of freedom and sample size of 35 participants considering Passing and Bablok regression analysis was deemed to be sufficient for this study [19, 25]. All analyses were performed using statistical software SPSS Version 23 (IBM) and MedCalc for Windows, version 19.4 (MedCalc Software, Ostend, Belgium). A p-value of <0.05 was considered statistically significant.

## Results

The mean age of the study participants was 42.4 ± 13 years. Most of the participants were male (65%), predominantly Caucasian/White (75%) and of Hispanic ethnicity (40%). More than one-third of the participants had coexisting medical conditions, and over half were on medications for the management of chronic diseases (Table 1).

**Table 1:** Demographic characteristics of the participants for the POCT i-STAT method and the DBC algorithm.

Characteristics	Frequency (%)
Age, years (Mean + SD; Range)	42.4 + 13.1; 18–64
Height, inches (Mean + SD; Range)	67.2 + 3.8; 60–74
Weight, pounds (Mean + SD; Range)	193.6 + 37.7; 122–285
**Gender**	
Male	24 (65)
Female	13 (35)
**Race**	
White	28 (76)
Black	8 (22)
Asian	1 (2.7)
**Ethnicity**	
Hispanic	15 (41)
Non-Hispanic	22 (59)
**Has co-morbidities**	13 (35)
**On medication for chronic diseases**	20 (54)
**Uses OTC products/natural supplements**	11 (30)

The results of both paired t-tests and Wilcoxon sign rank tests showed non-significant differences between the measurements obtained through the i-STAT and the DBC’s algorithm for parameters including pO2, pCO2, pH, HCO3, TCO2, and Cl (Table 2). These findings suggest an agreement between the i-STAT and DBC’s algorithm across a range of parameters, suggesting that the non-invasive transcutaneous approach of the DBC may offer a promising alternative to the invasive POCT approach. To further quantify this agreement, intraclass correlation coefficients (ICCs) were computed based on a single-rating, absolute-agreement, two-way mixed-effects model. The calculated ICCs for i-STAT and the DBC algorithm were as follows: 0.559 for Hb, 0.601 for pCO2, 0.105 for HCO3, 0.168 for TCO2, and 0.181 for Na. Considering the 95%CIs associated with these values, our results suggest a range of agreement levels: from poor to moderate absolute agreement between i-STAT and the DBC’s algorithm for Hb, pCO2, HCO3, TCO2, and Na, and weak agreement for pO2, HSO2, pH, K, Ca, and Cl (Table 3). The Passing and Bablok regression analyses demonstrated that values for Hb, pO2, pCO2, TCO2, Cl, and Na obtained from the i-STAT did not differ significantly when compared to those of the DBC’s algorithm. This suggests that, for these parameters, there were no apparent systematic or proportional differences between the two measurement methods, except for HCO3 (Table 3). These observations were further supported by the Passing and Bablok regression plots (S2 Fig).

**Table 2:** Comparison of analytes for agreement between iSTAT and DBC algorithm.

	i-STAT		DBC			
Analyte	Mean	SD	Mean	SD	t-statistics	p-value
Hb (%)	13.8	1.4	15.0	1.4	-5.01	< .001
pO_2_ (mmHg)	73.3	9.0	73.6	10.4	-0.11	0.916
pCo_2_ (mmHg)	39.6	3.4	40.4	4.2	-1.26	0.217
sO_2_	97.4	1.1	93.3	2.3	-5.13*	< .001
pH	7.4	0.0	7.4	0.1	-0.05	0.959
HCo_3_	25.5	1.7	25.5	5.1	-0.04	0.971
TCo_2_	26.7	1.8	26.7	5.1	-0.04	0.971
K^+^ (mmol/L)	3.8	0.3	4.4	0.5	-4.71*	< .001
Ca^++^ (mmol/L)	1.3	0.0	1.1	0.0	-5.31*	< .001
Cl^-^ (mmol/L)	103.5	2.0	102.7	1.5	1.61	0.116
Na^+^ (mmol/L)	137.9	2.0	141.1	1.5	-9.48	< .001

SD: Standard Deviation

*Wilcoxon sign rank test

**Table 3:** Intraclass Correlation Coefficient (ICC) and Passing and Bablok regression analysis of analytes measured using iSTAT and DBC algorithm.

	Intraclass Correlation Coefficient				Passing and Bablok Regression Analyses	
Analyte (unit)	(r)	95%CI	F ‐ test	p-value	Slope, 95%CI	Intercept, 95%CI
Hb (%)	0.559	0.001, 0.794	3.093	<0.001	0.9412 (0.5882, 1.4500)	1.8000 (-5.2850, 6.7000)
pO_2_ (mmHg)	-0.428	-1.879, 0.279	0.709	0.847	1.4443 (0.5963, 4.8362)	-31.6867 (-281.7376, 29.4164)
pCo_2_ (mmHg)	0.601	0.235, 0.794	2.532	0.003	1.5355 (0.9631, 2.3080)	-20.5790 (-51.9358, 2.8337)
sO_2_	-0.047	-0.235, 0.193	0.85	0.686	0.000 (0.000, 0.5000)	1.9900 (1.0050, 1.9900)
pH	-0.07	-1.138, 0.457	0.936	0.578	0.0634 (-0.0200, 0.2100)	-6.9457 (5.8489, 7.5608)
HCo_3_	0.105	-0.779, 0.545	1.115	0.373	6.4879 (3.0545, 30.5000)	-142.2167 (-760.8300, -53.3427)
TCo_2_	0.168	-0.651, 0.577	1.197	0.296	0.1636 (0.0000, 0.3160)	22.5730 (18.4344, 27.0000)
K^+^ (mmol/L)	-0.141	-0.521, 0.241	0.728	0.827	2.3667 (1.0000, 12.0000)	-0.7253 (-6.2900, 0.0700)
Ca^++^ (mmol/L)	-0.003	0.029, 0.044	0.946	0.565	0.0000 (0.0000, 0.0000)	0.0400 (0.0400, 0.0400)
Cl^-^ (mmol/L)	-0.301	-1.435, 0.317	0.759	0.794	0.5088 (0.1457, 1.4000)	50.2587 (-42.1500, 87.3900)
Na^+^ (mmol/L)	0.181	-0.168, 0.494	1.75	0.049	0.5925 (0.3650, 1.0650)	59.2725 (-5.8950, 90.6700)

r: Intraclass correlation coefficient; CI: Confidence interval; (r)*: Spearman correlation coefficient; Log transformed data was used for sO2, K and Ca for passing and Bablok regression analyses.

**Fig 1:**
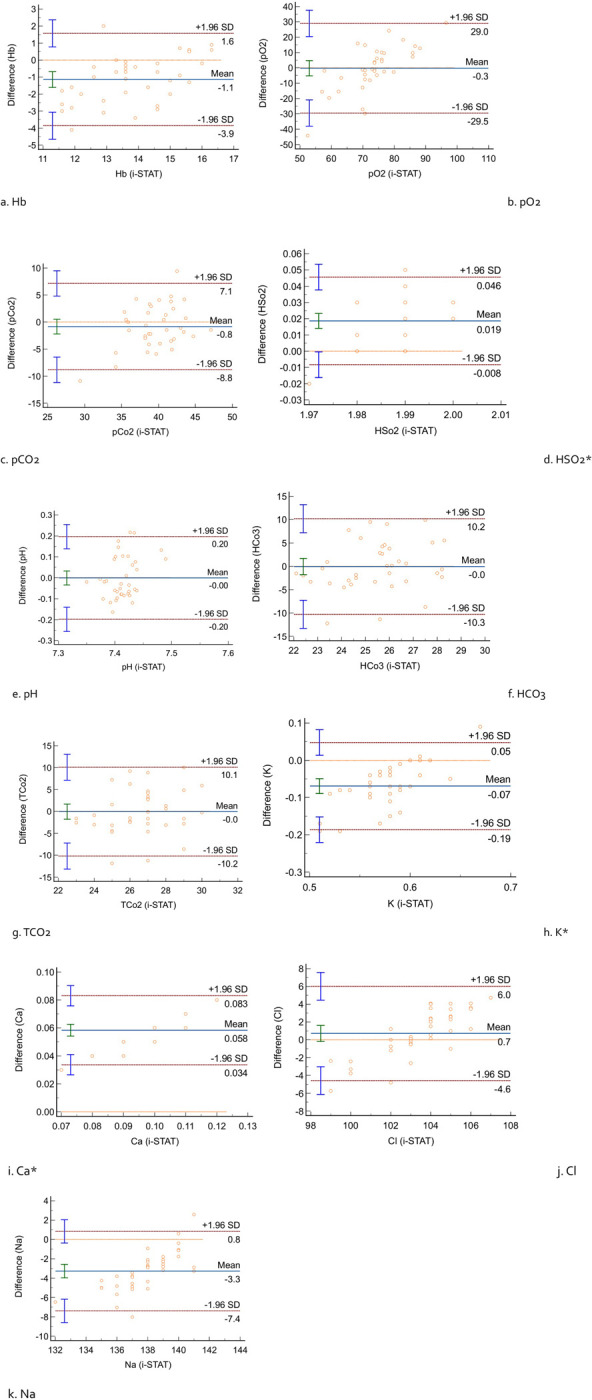
Comparison between i-STAT and DBC algorithm with Bland-Altman Plot (a) Hb; (b) pO2; (c) pCO2; (d) HSO2; (e) pH; (f) HCO3; (g) TCO2; (h) K; (i) Ca; (j) Cl; (k) Na; Y axis: Difference (i-STAT–DBC); *Log transformed data.

To illustrate the agreement between the values obtained from the DBC’s algorithm and the i-STAT (reference), Bland-Altman plots were generated (Fig 1). These plots explained the range of differences relative to the i-STAT reference values, encapsulating both systematic (bias) and random error (precision) within their limits of agreement. The limits of agreement estimate the interval in which a proportion of the differences between measurements is expected to fall, providing a valuable metric for comparing the likely discrepancies between individual results obtained from the two methods. It is evident that values for Hb, K, and Na measured by the DBC algorithm were slightly higher than those obtained by the i-STAT, indicating some systematic differences between these two methods. The mean difference between the i-STAT, reference standard and DBC algorithm for Hb was (-1.14/dl ± 1.39), pO2 (-0.26 mmHg ± 14.9), pCO2 (-0.84 mmHg ± 4.07), HSO2 (4.1% ± 2.67), pH (0.0 ± 0.10), HCO3 (-0.03 mmol/L ± 5.22), TCO2 (-0.03 mmol/L ± 5.18), K (-0.66 ± 0.59), Ca (0.16 mmol/L ± 9.03), Cl (0.71 mmol/L ± 2.70), and Na (-3.26 mmol/L ± 2.09). From the Bland-Altman plots, the 95% limits of agreement ranged from -0.38 to 1.58 for Hb, -29.50 to 28.98 for pO2, -8.83 to 7.15 for pCO2, -0.008 to 0.046 for HSO2, -0.20 to 0.20 for pH, -10.27 to 10.21 for HCO3, -10.17 to 10.11 for TCO2, -0.19 to 0.05 for K, 0.034 to 0.083 for Ca, -4.58 to 6.01 for Cl, and -7.38 to 0.84 for Na. Considering the Bland-Altman plots, most paired data points fell within ±1.96 standard deviations (SD), corresponding to the upper and lower limits of agreement (Table 4). The most substantial deviation between the i-STAT and the DBC’s algorithm was observed for pO2, a parameter that significantly influences the accuracy of calculated values.

**Table 4:** Results of method comparison on Bland and Altman plot (i-STAT considered as a reference standard).

	Mean Difference (Bias)	SD	LLA	ULA
Hb (%)	-1.14	1.39	-3.86	1.58
pO2 (mmHg)	-0.26	14.92	-29.50	28.98
pCo2 (mmHg)	-0.84	4.08	-8.83	7.15
HSo2*	0.019	2.67	-0.008	0.046
pH	0.00	0.10	-0.20	0.20
HCo3	-0.03	5.22	-10.27	10.21
TCo2	-0.03	5.18	-10.17	10.11
K+ (mmol/L)*	-0.07	0.60	-0.19	0.05
Ca2+ (mmol/L)*	0.058	0.04	0.034	0.083
Cl- (mmol/L)	0.71	2.70	-4.58	6.01
Na+ (mmol/L)	-3.27	2.10	-7.38	0.84

SD: Standard Deviation; LLA: Lower Limit of Agreement; ULA: Upper Limit of Agreement

*Log transformed data

A comparison of responses to the comfort level questionnaire showed that the DBC non-invasive algorithm was significantly more comfortable for participants compared to the invasive i-STAT method (Table 5). Participants expressed lower discomfort scores across all six domains of the questionnaire, including emotional state, uneasiness with attachment, perception of harm, perceived changes, movement constraints, and anxiety level, when utilizing the DBC’s non-invasive algorithm as opposed to the i-STAT invasive method.

**Table 5:** Comparison of participants’ comfort level with use of both methods.

Items	i-STAT	DBC	p-value
Q1. I am worried about how I look when I wear this device. I feel tense or on edge because I am wearing the device (Emotional state).	2.297	0.338	0.002
Q2. I can feel the device on my body. I can feel the device moving (Uneasiness with Attachment).	3.054	0.392	<0.001
Q3. The device is causing me some harm. The device is painful to wear (Perception of harm).	4.189	0.149	<0.001
Q4: Wearing the device makes me feel physically different. I feel strange wearing the device (Perceived changes).	2.230	0.189	0.001
Q5: The device affects the way I move. The device inhibits or restricts my movement (Movement constraints).	2.689	0.649	0.002
Q6: I do not feel secure wearing the device (Anxiety level).	2.392	0.243	<0.001

Both i-STAT and DBC values are presented as a mean score

## Discussion

In this study measurements obtained from two distinct approaches for a set of blood values were compared. To our knowledge, this is the first study that compared invasive POCT approach with that of non-invasive algorithm for blood gas measurement. Despite the rising popularity of invasive POCT devices due to their ease of use, they still present significant challenges such as chances of injury, infection, and delays in obtaining results [26, 27].

Therefore, DBC’s non-invasive approach appears to be promising, offering a practical and user-friendly alternative. The ideal non-invasive method for blood gas measurement should be practical, portable, inexpensive, painless, and user-friendly, while aligning closely with established invasive reference standards [28]. In this study involving healthy participants, all values fell within the normal range except for pO2 and pCO2, which were outside the normal range for both DBC and the i-STAT, a reference standard. However, overall, the non-invasive DBC algorithm showed good agreement with i-STAT for various blood parameters including pO2, pCO2, pH, HCO3, TCO2, and Cl in paired comparisons.

The intraclass correlation coefficients between these two approaches, analyzed using a two-way mixed-effects model, indicated moderate to weak agreement. However, it’s essential to assess agreement based on the 95%CI rather than just the ICC estimate obtained from a reliability study. The 95%CI for a set of blood parameters revealed moderate agreement for Hb, pCO2, HCO3, TCO2, and Na, while indicating weak agreement for pO2, HSO2, pH, K, Ca, and Cl. The moderate to low ICC values might have stemmed from the study’s limited number of participants, leading to greater variability among them. The Bablok-Passing regression method assumes a linear relationship, expecting residuals to exhibit a random pattern close to a normal distribution. Typically, 95% of residuals should fall within ± 1.96 times the residual standard deviation, signifying random differences between measurement approaches. When the intercept is near ’0’ and slope near ’1’, the residual plot mirrors the Bland Altman plot, allowing for similar interpretation [19]. In this study, Passing and Bablok regression analyses revealed no systematic or proportional differences between approaches for most blood values, except HCO3. The 95% confidence intervals for intercepts and slopes across measured parameters included ’0’ and ’1’. Correspondingly, the regression plots supported these findings, indicating good agreement for Hb, pO2, pCO2, TCO2, Cl, and Na. These results suggest no significant difference between the approaches, supporting their interchangeability.

Validation of clinical measurements involves demonstrating reliability and reproducibility for the intended use, acknowledging inherent measurement errors. When comparing methods, neither provides an absolute correct measurement, underscoring the need to assess their agreement [29]. Correlation studies evaluate relationships between variables, not differences, thus aren’t recommended for method comparability assessment. Bland and Altman introduced an approach assessing agreement by studying mean differences and constructing limits of agreement [21, 22]. In this study, Bland-Altman plots revealed slightly higher values for Hb, K, and Na with the DBC algorithm compared to i-STAT, indicating systematic differences. Similarly, pO2 and pCO2 values were higher with the DBC algorithm. However, for HSO2, pH, HCO3, TCO2, K, and Ca, the bias between non-invasive DBC and invasive i-STAT approaches was nearly zero. Transcutaneous O2 measurements are commonly lower due to inherent biological variability in dermal perfusion and oxygen delivery, as noted in study by Blake et al. [30]. with healthy volunteers exhibiting varied pO2 levels across different body parts. This variability challenges the establishment of narrow ’normal’ values.

Most paired data points on the Bland-Altman plots for the blood values in this study fell within ±1.96 standard deviations, within the upper and lower limits of agreement. Notably, there was significant deviation for pO2, impacting the accuracy of derived values. Despite differences observed in parameters using the DBC algorithm, they were clinically insignificant for Hb, pH, K, and Ca when compared against recommended clinical limits by [24, 31]. While the Bland-Altman plot identifies bias and an agreement range, encompassing 95% of measurement differences, it doesn’t determine whether the agreement is sufficient for interchangeable method use. The adequacy of the agreement interval relies on specific analytical, biological, or clinical goals, defining whether the interval is suitably narrow or too wide for the intended purpose. The deviations observed may imply a lack of precision in the DBC algorithm, potentially stemming from small participant numbers or the need for a correction factor to address systematic errors. Alternatively, a more robust transcutaneous pO2 measurement protocol could reduce variation, aligning values closer to those observed with the i-STAT reference standard.

Interestingly, participants in this study reported significantly higher comfort levels with the non-invasive DBC approach compared to the invasive i-STAT method. They expressed ease, reduced anxiety, and comfort with sensor attachment, noting fewer restrictions on movement with the DBC algorithm. These findings highlight a greater acceptability for non-invasive approaches, encouraging the future development of improved non-invasive POCT devices. It’s essential to note that this pilot study primarily aimed to assess agreement between the DBC algorithm and i-STAT for blood values and didn’t investigate the clinical significance of observed values, given the inclusion of solely healthy individuals.

Both measurement approaches were efficient and user-friendly. The invasive i-STAT devices took on an average 30 minutes for measurement, while the noninvasive DBC approach took 10 minutes. The i-STAT approach, involving blood drawing, showed varied durations among participants and required a phlebotomist, making it take a relatively longer time. It also presented drawbacks such as pain, anxiety, injury, or infection risks. Factors like poor peripheral circulation or difficulties in blood collection also could have affected its accuracy. On the other hand, the non-invasive DBC method had no risk of injury or infection and was less time-consuming. However, it could be influenced by factors like skin conditions, texture, sensor calibration, or fragility.

There are several limitations to acknowledge in our study. Since the primary focus of this pilot investigation was methodological refinement and validation of the DBC algorithm and software, we did not include ill subjects to protect them from any potential harm of using both devices. A small sample of only healthy individuals were included in this initial testing phase which has limited our exploration of variations within input values.

Moreover, our findings indicated that transcutaneous measurements of pO2 and pCO2 contributed notably to high standard deviations, possibly due to imprecise measurements or errors from input devices or the DBC algorithm itself. The DBC algorithm relied on input values from Masimo Rad and TINA TCM4. The accuracy of pO2 measurements can be affected by issues like improper calibration, sensor positioning, or lack of maintenance. Additionally, using forehead temperature measurements, while convenient, might introduce variability due to its inherent lack of precision, especially when temperature strongly influences the results. Several studies have reported a systematic overestimation of oxygen saturation on POCT devices among individuals with skin of darker pigmentation compared with individuals with lighter [32]. Characteristics of the subjects including skin color or race/ethnicity could have influenced our results. Small sample had limited equal representations of such groups in this study and ascertaining their influence on our study results.

## Conclusions

The DBC algorithm and software exhibits robustness and reliability in analyzing some hematological parameters and electrolytes among healthy subjects, although requiring precise input values for accurate and clinically relevant results. In addition, participants reported a higher level of comfort when using the DBC algorithm and software compared to the invasive i-STAT approach. However, it remains to be determined whether these findings hold broader clinical significance, particularly among critically ill adults and children, where the implications of this non-invasive approach may be more substantial. Further research and evaluation are warranted to ascertain its applicability in diverse clinical scenarios.

## Supporting information

S1 Fig Comfort level questionnaire for study participants (Adapted from: Knight, J.; Baber, C.; Schwirtz, A.; Bristow, H. The comfort assessment of wearable computers. In Proceedings of the IEEE Sixth International Symposium on Wearable Computers, White Plains, NY, USA, 21–23 October 2003; Volume 2, pp. 65–74).
(PDF)

S2 Fig Passing and Bablok Regression Plots (a) Hb; (b) pO2; (c) pCO2; (d) HSO2; (e) pH; (f) HCO3; (g) TCO2; (h) K; (i) Ca; (j) Cl; (k) Na; Blue line: Regression line; Dashed line: Identity line; *Log transformed data.
(PDF)
