# Supplementary material for: Evaluation of agreement between a noninvasive method for real-time measurement of critical blood values with a standard point-of-care device
Source: PLoS One. 2024 Jun 18;19(6):e0304706. doi: 10.1371/journal.pone.0304706 (PMC11185484; doi:10.1371/journal.pone.0304706)
Supplement: S1 Fig — The comfort assessment of wearable computers. In Proceedings of the IEEE Sixth International Symposium on Wearable Computers, White Plains, NY, USA, 21–23 October 2003; Volume 2, pp. 65–74). (PDF) [file pone.0304706.s001.pdf]

Emotion Low/High
I am worried about how I look when I wear this device. I feel tense or on edge because I am wearing the device.

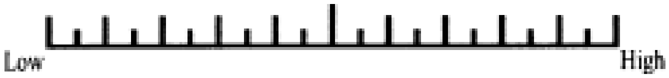

Attachment Low/High
I can feel the device on my body. I can feel the device moving.

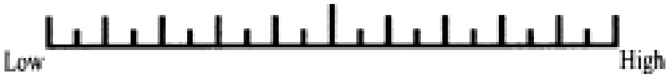

Harm Low/High
The device is causing me some harm. The device is painful to wear.

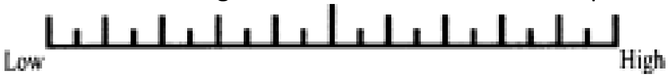

Perceived change Low/High
Wearing the device makes me feel physically different. I feel strange wearing the device.

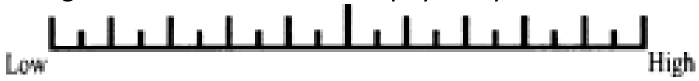

Movement Low/High
The device affects the way I move. The device inhibits or restricts my movement.

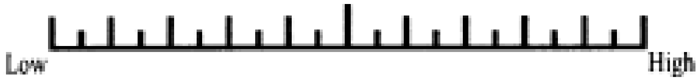

Anxiety Low/High
I do not feel secure wearing the device.

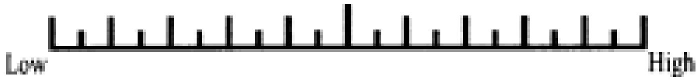

1. Can you tell me which device you preferred and why?
2. Is there anything else you want to provide about the DBC-NI device you would like to see?

Supplemental Fig 1. Comfort level questionnaire for study participants (Adapted from: Knight, J.; Baber, C.; Schwirtz, A.; Bristow, H. The comfort assessment of wearable computers. In Proceedings of the IEEE Sixth International Symposium on Wearable Computers, White Plains, NY, USA, 21–23 October 2003; Volume 2, pp. 65–7
