# Supplementary material for: Evaluation of agreement between a noninvasive method for real-time measurement of critical blood values with a standard point-of-care device
Source: PLoS One. 2024 Jun 18;19(6):e0304706. doi: 10.1371/journal.pone.0304706 (PMC11185484; doi:10.1371/journal.pone.0304706)
Supplement: S2 Fig — Passing and Bablok Regression Plots (a) Hb; (b) pO2; (c) pCO2; (d) HSO2; (e) pH; (f) HCO3; (g) TCO2; (h) K; (i) Ca; (j) Cl; (k) Na; Blue line: Regression line; Dashed line: Identity line; *Log transformed data. (PDF) [file pone.0304706.s002.pdf]

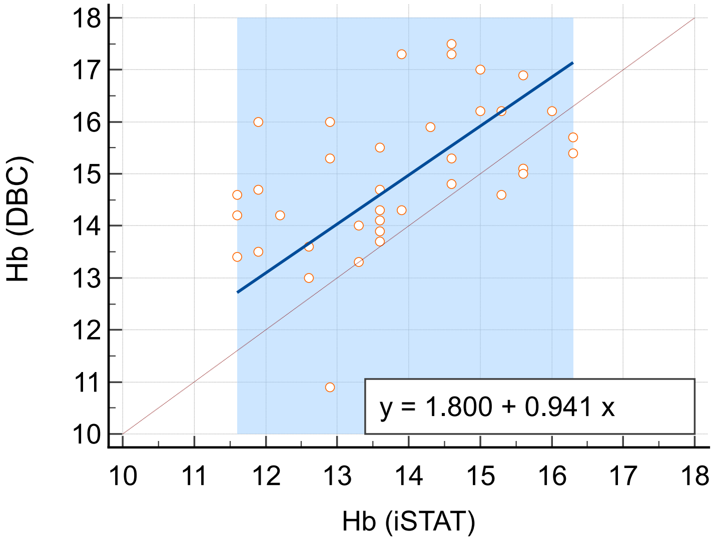

a. Hb

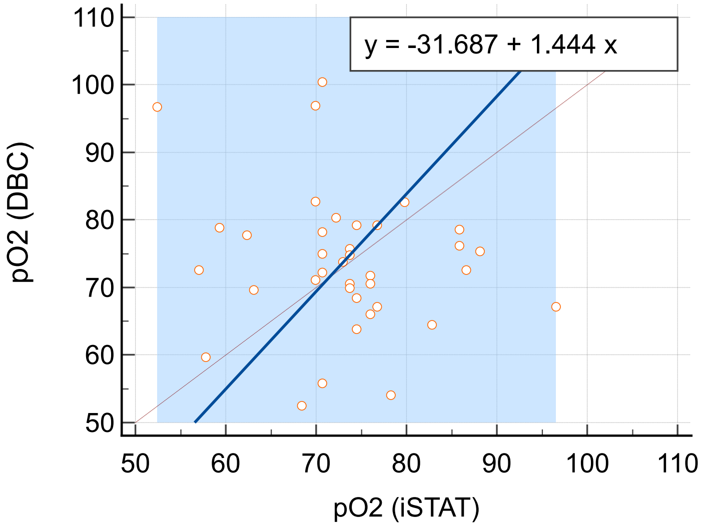

b. pO2

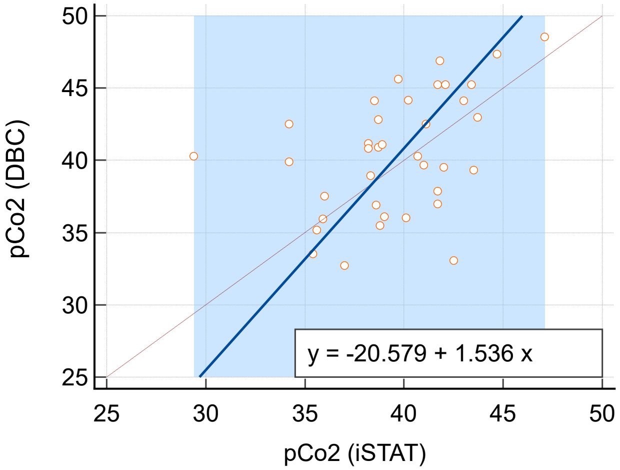

c. pCO2

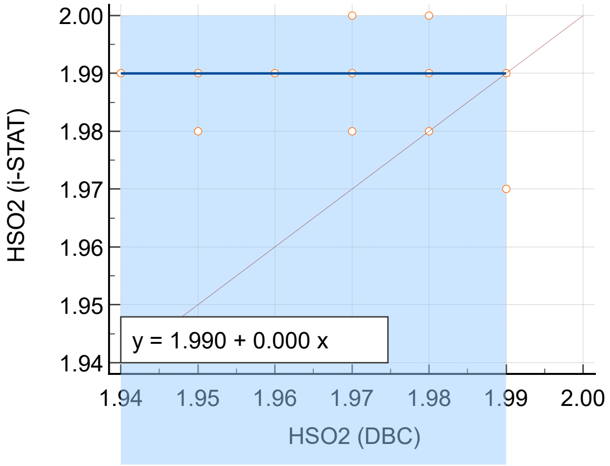

d. HSO2*

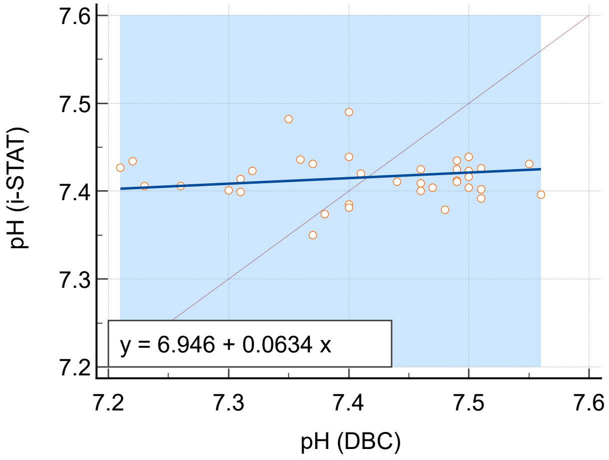

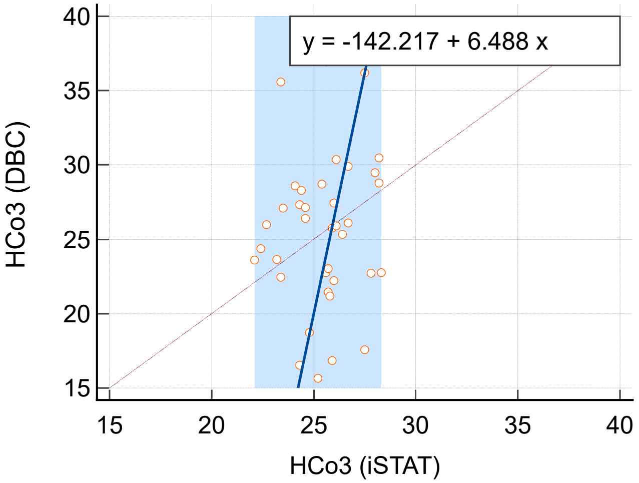

e. pH

f. HCO3

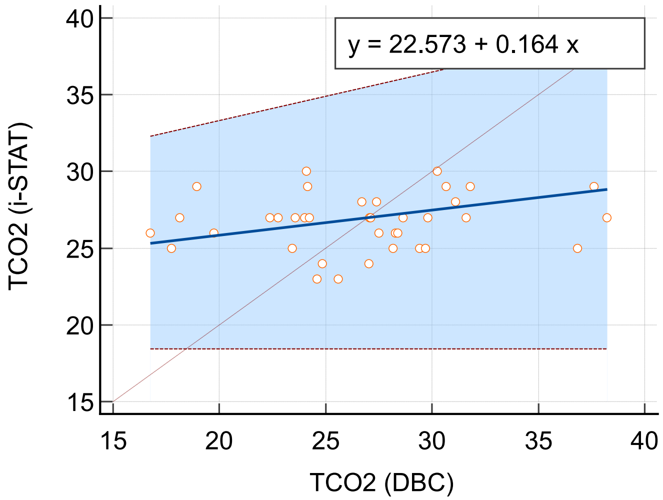

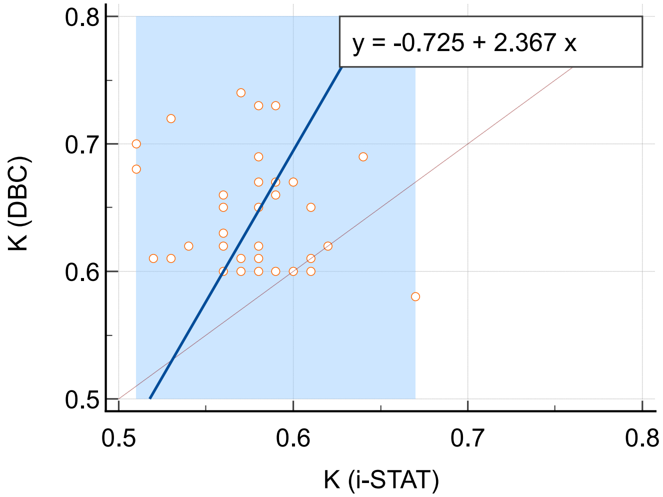

g. TCO2

h. K*

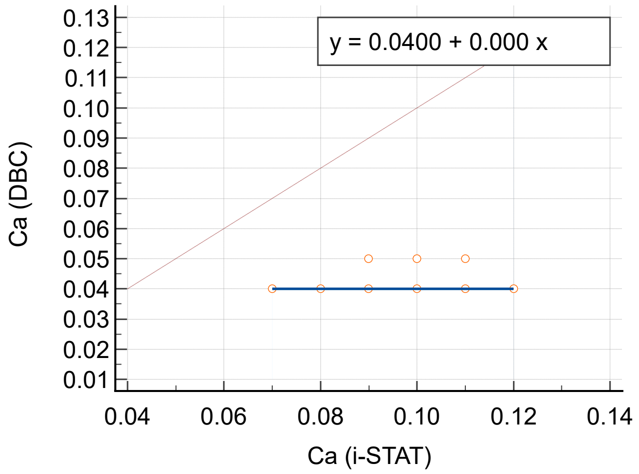

i. Ca*

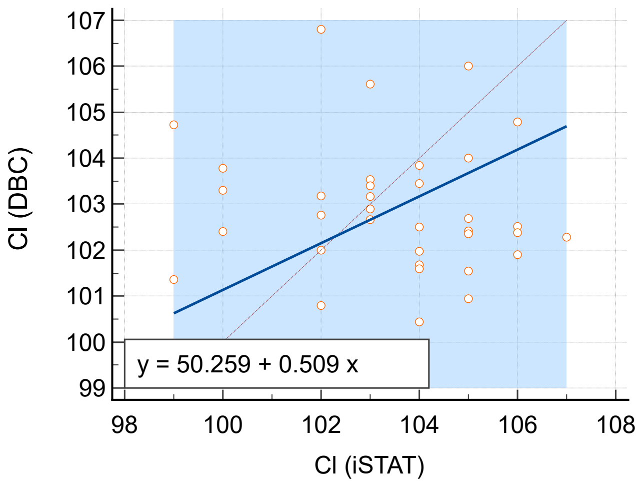

j. Cl

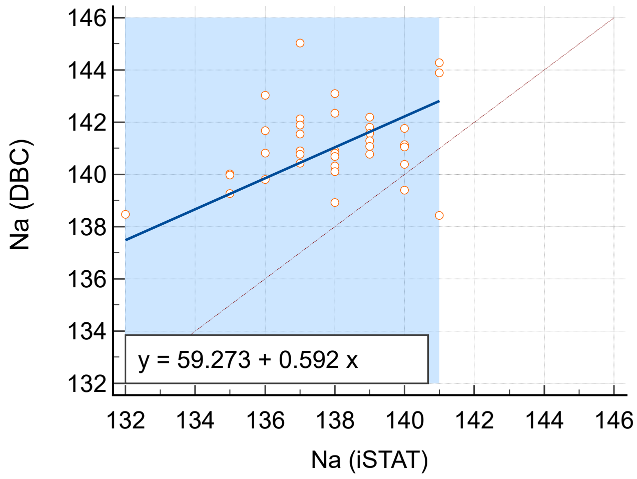

k. Na

Supplemental Fig 2. Passing and Bablok Regression Plots (a) Hb; (b) pO_2; (c) pCO_2; (d) HSO_2; (e) pH; (f) HCO_3; (g) TCO_2; (h) K; (i) Ca; (j) Cl; (k) Na; Blue line: Regression line; Dashed line: Identity line; *Log transformed data.
